# S100A8/A9 mediate the reprograming of normal mammary epithelial cells induced by dynamic cell–cell interactions with adjacent breast cancer cells

**DOI:** 10.1038/s41598-020-80625-2

**Published:** 2021-01-14

**Authors:** Seol Hwa Jo, Woo Hang Heo, Hye-Youn Son, Mingji Quan, Bok Sil Hong, Ju Hee Kim, Han-Byoel Lee, Wonshik Han, Yeonju Park, Dong-Sup Lee, Nam Hoon Kwon, Min Chul Park, Jeesoo Chae, Jong-Il Kim, Dong-Young Noh, Hyeong-Gon Moon

**Affiliations:** 1 Interdisciplinary Graduate Program in Cancer Biology, Seoul National University College of Medicine, Seoul, Korea; 2 Center for Medical Innovation, Biomedical Research Institute, Seoul National University Hospital, Seoul, Korea; 3 Department of Surgery, Seoul National University College of Medicine, 103 Daehak-ro, Jongno-gu, Seoul, 03080 Korea; 4 Medical Research Center, Genomic Medicine Institute, Seoul National University College of Medicine, Seoul, Korea; 5 Department of Biomedical Sciences, Seoul National University College of Medicine, Seoul, Korea; 6 Medicinal Bioconvergence Research Center, Seoul National University, Suwon, Korea; 7 Department of Biochemistry, Seoul National University College of Medicine, Seoul, Korea; 8 Cancer Research Institute, Seoul National University College of Medicine, Seoul, Korea

**Keywords:** Cancer, Cell biology

## Abstract

To understand the potential effects of cancer cells on surrounding normal mammary epithelial cells, we performed direct co-culture of non-tumorigenic mammary epithelial MCF10A cells and various breast cancer cells. Firstly, we observed dynamic cell–cell interactions between the MCF10A cells and breast cancer cells including lamellipodia or nanotube-like contacts and transfer of extracellular vesicles. Co-cultured MCF10A cells exhibited features of epithelial-mesenchymal transition, and showed increased capacity of cell proliferation, migration, colony formation, and 3-dimensional sphere formation. Direct co-culture showed most distinct phenotype changes in MCF10A cells followed by conditioned media treatment and indirect co-culture. Transcriptome analysis and phosphor-protein array suggested that several cancer-related pathways are significantly dysregulated in MCF10A cells after the direct co-culture with breast cancer cells. S100A8 and S100A9 showed distinct up-regulation in the co-cultured MCF10A cells and their microenvironmental upregulation was also observed in the orthotropic xenograft of syngeneic mouse mammary tumors. When S100A8/A9 overexpression was induced in MCF10A cells, the cells showed phenotypic features of directly co-cultured MCF10A cells in terms of in vitro cell behaviors and signaling activities suggesting a S100A8/A9-mediated transition program in non-tumorigenic epithelial cells. This study suggests the possibility of dynamic cell–cell interactions between non-tumorigenic mammary epithelial cells and breast cancer cells that could lead to a substantial transition in molecular and functional characteristics of mammary epithelial cells.

## Introduction

In solid tumors, the complex tumor microenvironment controls all steps of tumor progression and metastasis^1,2^. The tumor microenvironment is comprised of various endogenous and recruited cells that undergo dynamic cell–cell interactions with malignant epithelial cells and contribute to the tumor cell’s behaviors^3,4^. For example, cancer-associated fibroblasts actively remodel extracellular matrix and immune microenvironment, and cancer-associated adipocytes provide inflammatory milieu that support tumor growth^5,6^. Moreover, recent efforts to target the immune microenvironment have shown promising therapeutic responses in selected solid tumors^7^. Therefore, understanding the molecular mechanisms of the tumor-microenvironment interactions can provide scientific basis for developing novel therapeutic strategies that target the tumor microenvironment^3,8,9^.

Normal epithelial cells are closest neighbors to the malignant transformed cells in human epithelial tumors arising from solid organs. During the early steps of carcinogenesis, the normal epithelial cells may exert tumor-suppressive effects by promoting protrusion of transformed epithelial cells from the epithelial layers^10–12^. However, the tumor-suppressive effects of normal epithelial cells may not last throughout the solid tumor progression. While the normal myoepithelial cells obtained from healthy human breast tissues contribute to the maintaining polarity of mammary epithelial cells and suppress aberrant growth, the myoepithelial cells derived from breast cancer tissues failed to restore physiologic polarity in mammary epithelial cells and showed increased expression of various chemokines such as CXCL12^13,14^. These reports suggest a potential functional transition of normal epithelial cells caused by adjacent malignant epithelial cells which may contribute the progression of solid tumors.

In this study, we show that breast cancer cells and non-tumorigenic mammary epithelial cells undergo dynamic cell–cell interactions that lead to a substantial reprograming of molecular characteristics of the mammary epithelial cells. The reprograming of normal mammary epithelial cells includes phenotypes changes as well as dysregulations of mRNA expression and cell signaling activities. Our data suggests that S100A8/A9 upregulation in non-tumorigenic mammary epithelial cells may play a critical role in the phenotype shifting induced by adjacent cancer cells.

## Results

### Dynamic interaction between breast cancer cells and non-transformed mammary epithelial cells

**Figure 1:**
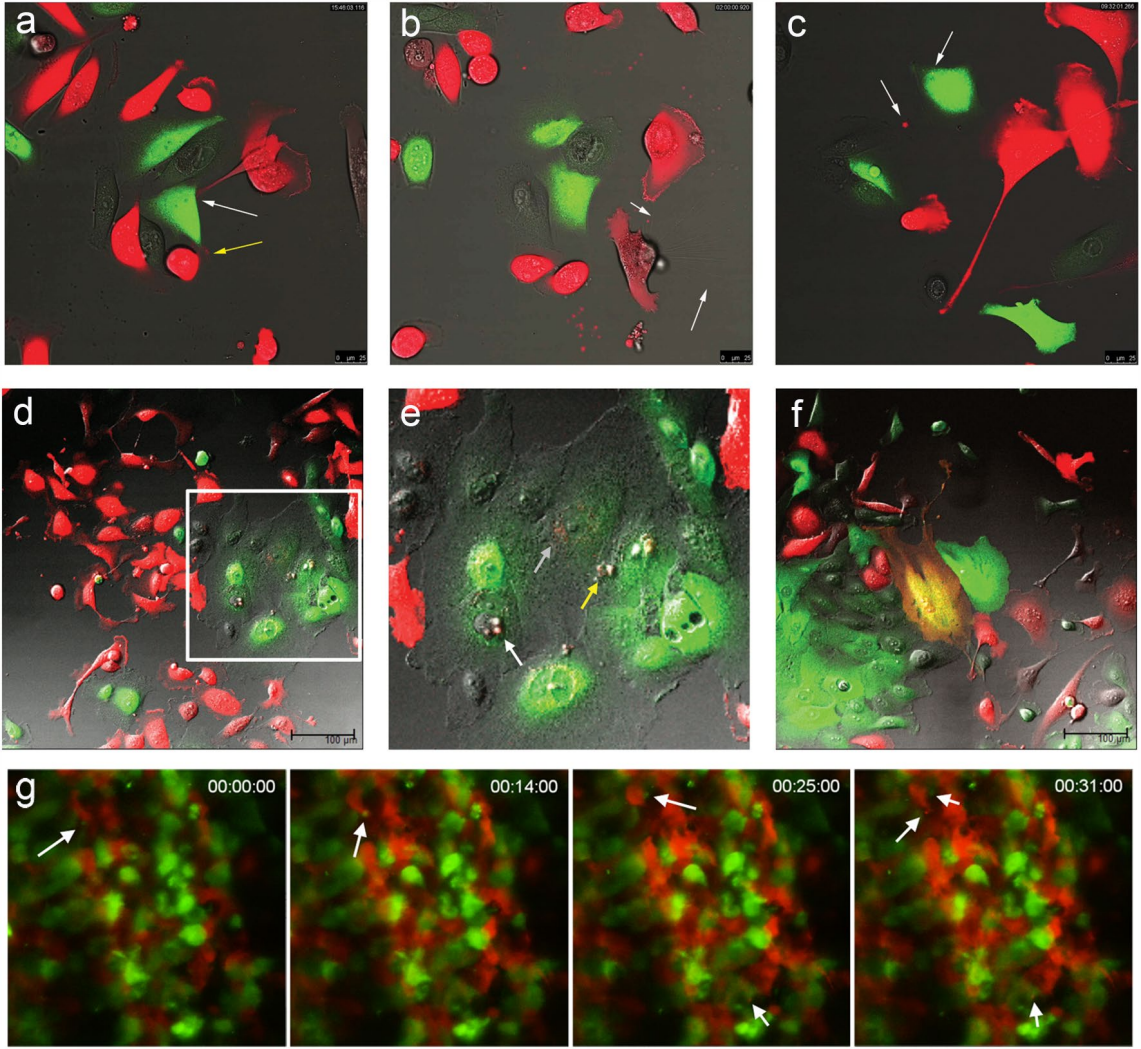
Dynamic cell–cell interactions between breast cancer cells and non-tumorigenic mammary epithelial cells. Representative images of cell–cell interactions between RFP-transfected MDA-MB-231 breast cancer cells and GFP-transfected MCF10A non-tumorigenic mammary epithelial cells were captured using time-lapse confocal microscopy (**a**–**d**). (**a**) MDA-MB-231 cells use lamellipodia-like structures to contacting MCF10A cells (yellow arrow) or distant MCF10A cells (white arrow). (**b**) Nanotube-like structures extending from MDA-MB-231 cells are seen (white arrows). (**c**) RFP-expressing vesicles from MDA-MB-231 cells are transferred to MCF10A cells (white arrows). (**d**,**e**) The area within the white rectangle in (**d**) is shown in (**e**). Transferred vesicles from MDA-MB-231 cells are located in both nucleus (white arrow) and cytoplasm (yellow arrow) of MCF10A cells. (**f**) A minority of co-cultured cells show dual fluorescence. (**g**) The representative images of the in vivo behavior of MDA-MB-231 cells and MCF10A cells co-injected in the earlobe of mouse are shown with migrating extracellular vesicles (white arrows).

First, we determined the presence and the extent of cell–cell interactions in vitro between the breast cancer cells and mammary epithelial cells. We co-cultured the RFP-transfected breast cancer cells (MDA-MB-231) with GFP-transfected non-transformed mammary epithelial cells (MCF10A) using in vitro direct co-culture method. While the majority of MCF10A cells maintained the clusters of adherent cells, MDA-MB-231 cells showed spreading patterns of cell growth and the cells infiltrated between the MCF10A cell clusters (Supplementary Fig. S1a). The time-lapse imaging of the cells showed that MDA-MB-231 cells had more frequent cell movements than the MCF10A cells and the cells showed various dynamic cell–cell interaction patterns (Supplementary Videos S1 and S2). MDA-MB-231 cells formed both lamellipodia-like structures for adjacent cells and nanotube-like projections for long-range cell–cell interactions (Fig. 1a,b, Supplementary Video S1)^15,16^. The lamellipodia-like structures of MDA-MB-231 cells actively contacted the MCF10A cells and a portion of extended lamellipodia could remain as extracellular vesicles which were then engulfed by adjacent MCF10A cells (Fig. 1c, Supplementary Video S2). Overall, the physical interactions between the MDA-MB-231 and MCF10A cells occurred less than 1% of the time (Supplementary Fig. S1b). Among the various cell–cell interactions, the exchanges of extracellular vesicles were frequently observed. MCF10A cells engulfed extracellular vesicles originated from MDA-MB-231 cells, and the vesicles were often transferred to the nucleus while some stayed at cytoplasm (Fig. 1d,e). Additionally, a small proportion of co-cultured cells (1–2%) exhibited mixed fluorescence (Fig. 1f, Supplementary Fig. S1c).

To observe the cell–cell interactions in vivo, we inoculated a mixture of MDA-MB-231 cells and MCF10A cells in the earlobes of nude mouse and obtained the time-lapse imaging data. After one hour of the inoculation, the movements of the MDA-MB-231 cells around the MCF10A cells were detectable (Supplementary Video S3). When compared to the in vitro co-culture condition, the in vivo co-injection resulted in more frequent cell to cell interaction (Supplementary Fig. S1d). The incidence of the cell–cell interactions and exchange of vesicles were more frequently observed at 24 h after the injection (Fig. 1g, Supplementary Video S4, Supplementary Fig. S1d). These data suggest that the cancer cells and epithelial cells can undergo a dynamic range of physical contacts and cell–cell interactions both in vitro and in vivo.

### Phenotype changes observed in the MCF10A cells when co-cultured with breast cancer cells

We then determined whether the cell–cell interactions with breast cancer cells induce phenotype changes in MCF10A cells. First, we co-cultured MCF10A cells with breast cancer cell lines using in-direct co-culture system. MCF10A cells co-cultured indirectly with cancer cells showed no significant changes in cell morphology, in cell growth rates, and in migration capacity (Supplementary Fig. S2a–c). Interestingly, indirect co-culture with MDA-MB-231 cells resulted in up-regulation of E-cadherin gene in MCF10A cells (Supplementary Fig. S2d). Additionally, indirect co-culture did not affect the cell growth in matrigel (Supplementary Fig. S2e). However, the indirect co-culture with MDA-MB-231 cells resulted in an increase of colony formation of MCF10A cells (Supplementary Fig. S2f).

**Figure 2:**
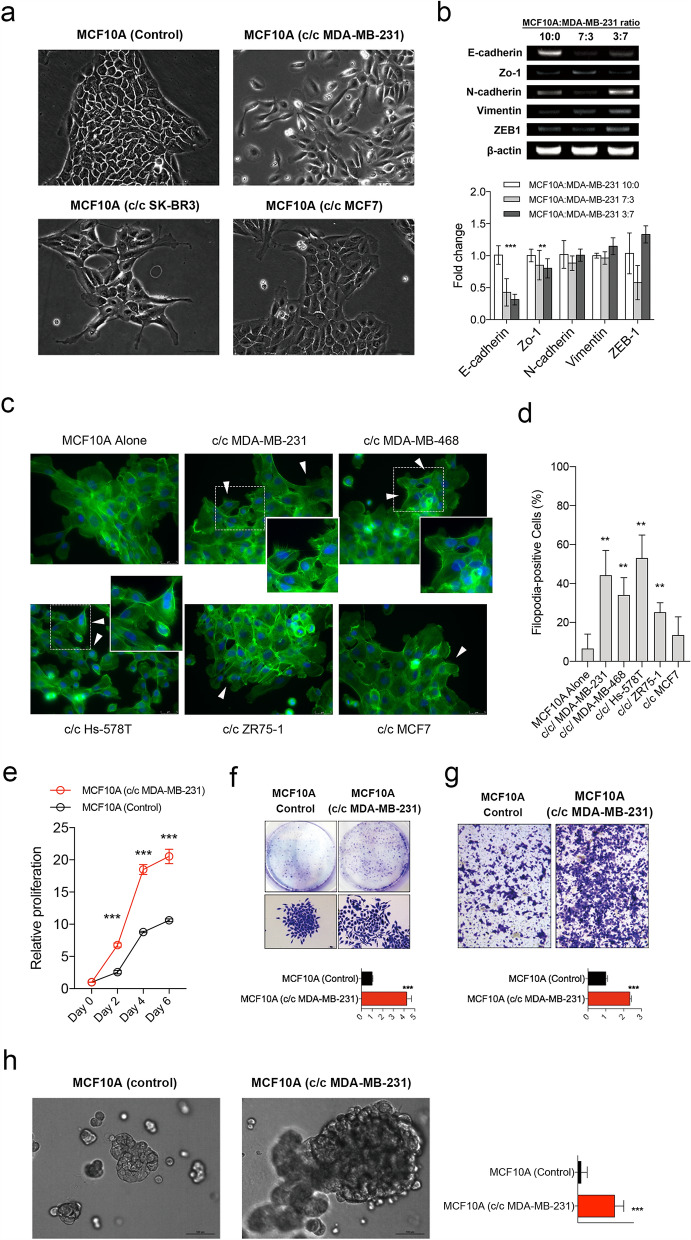
Phenotype transition of non-tumorigenic mammary epithelial cells when directly co-cultured with breast cancer cells. (**a**) MCF10A cells were directly co-cultured with MDA-MB-231, SK-BR3, and MCF7 breast cancer cells. After sorting out the MCF10A cells, the cellular morphologies are shown. (**b**) The expression levels of epithelial-mesenchymal transition markers were measured by RT-PCR in MCF10A cells after co-culture with MDA-MB-231 cells (upper) and quantified (lower). Full-length gels are presented in Supplementary Fig. S6. (**c**) The result of F-actin staining in MCF10A cells were observed after direct co-culture associated with various breast cancer cells (MDA-MB-231, MDA-MB-468, Hs-578 T, ZR75-1, and MCF7). (**d**) Quantification of filopodia (cell protrusions) in MCF10A cells were revealed after direct co-culture. (**e**) Relative growth rate of MCF10A cells co-cultured with MDA-MB-231 was measured based on ATP level with CellTiter-Glo reagent. The representative images and quantified results of the colony-formation assay (**f**), the transwell migration assay (**g**), the 3-dimensional matrigel culture assay (**h**) for co-cultured MCF10A cells with MDA-MB-231 cells are shown. Error bars denote mean ± SD. *P < 0.05, **P < 0.01, ***P < 0.001. P values are determined by the Mann–Whitney test.

In contrast, when the MCF10A cells were cultured in the direct co-culture system with breast cancer cells, the MCF10A cells showed substantial phenotypic changes. Directly co-cultured MCF10A cells showed changes in cell morphology such as transition into spindle-shaped cells and loss of cell–cell adhesions (Fig. 2a). Additionally, the MCF10A cells showed marked decrease in E-cadherin expression when they were directly co-cultured with MDA-MB-231 cells (Fig. 2b, Supplementary Fig. S3a). When the MCF10A cells were stained against F-actin, we observed increased incidence of lamellipodia-like protrusions after they were co-cultured with breast cancer cells (Fig. 2c). Interestingly, triple negative breast cancer cells such as MDA-MB-231, MDA-MB-468, and Hs-578T caused more cell protrusions in MCF10A cells compared to the breast cancer cells of other subtypes (Fig. 2d). When the isolated MCF10A cells were grown in vitro, the proliferation rate, colony formation capacity, and the cell migration ability were significantly enhanced in cells directly co-cultured with MDA-MB-231 cells (Fig. 2e–g). Co-cultured MCF10A cells also showed higher numbers and larger sizes of cell spheres when they were grown in 3D matrigel (Fig. 2h).

Next, we investigated the conditioned media from breast cancer cells would also induce changes in MCF10A cells similar to those of the direct co-culture. The conditioned media from MDA-MB-231 cells showed no effect on cell morphology and EMT marker expression in MCF10A cells (Supplementary Fig. S3b,c). However, the conditioned media increased cell motility and resistance to serum starvation (Supplementary Fig. S3d,e). Interestingly, when the MCF10A cells were treated with the purified extracellular vesicles of MDA-MB-231 cells, the proliferation of MCF10A cells increased significantly (Supplementary Fig. S3f) while the cell motility was not affected (Supplementary Fig. S3g).

Taken together, our data suggest that breast cancer cells induce substantial and comprehensive phenotypic changes in non-tumorigenic MCF10A cells especially when they are in a direct co-culture system where cell to cell physical interactions are possible (Supplementary Fig. S3h).

### Gene expression changes and signaling dysregulation occurring in the co-cultured MCF10A cells reflect tumor microenvironment

**Figure 3:**
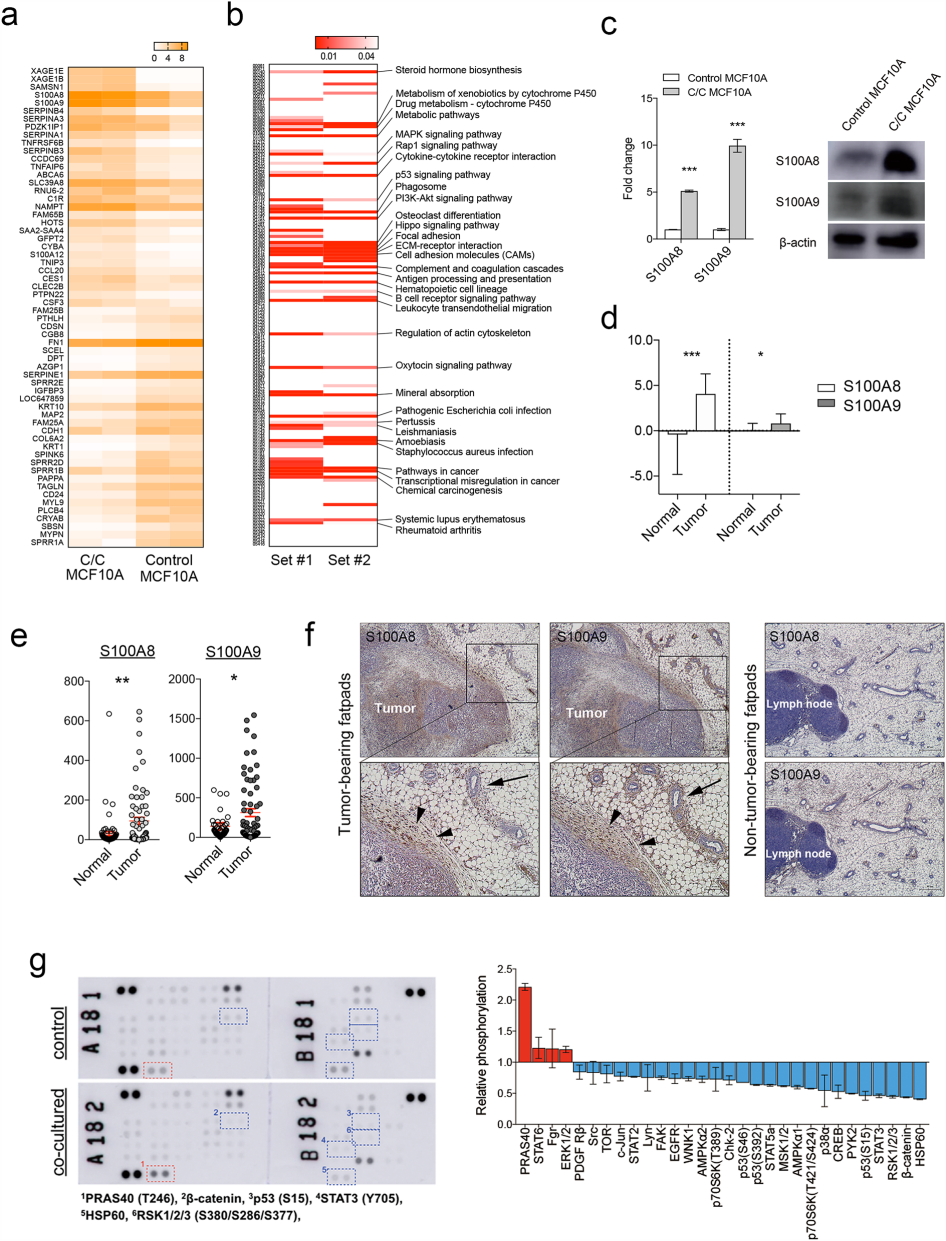
Transcriptomic and proteomic profiles of non-tumorigenic mammary epithelial cells co-cultured with breast cancer cells. (**a**) Heatmap shows the top 60 genes that were up- or down-regulated in MCF10A cells co-cultured with MDA-MB-231 cells. (**b**) Heatmap shows the significant enrichment of KEGG pathways based on the RNA sequencing data. The heatmap represents significant pathways identified from the pathway analysis using repeated RNA sequencing (comparison set #1 and set #2). Pathways significantly dysregulated in both datasets are listed on the right side. (**c**) S100A8/A9 mRNA levels and protein levels in MCF10A cells co-cultured with MDA-MB-231 cells are shown. Full-length blots are presented in Supplementary Fig. S6. (**d**) S100A8/A9 mRNA levels in breast cancer stromal tissues are shown (Finak et al. GSE9014). (**e**) The S100A8/A9 mRNA levels obtained from human breast cancer RNA sequencing data are shown. (**f**) On left, mouse mammary fatpad tissues bearing 4T1 breast cancer cells were stained against S100A8 and S100A9. Lower panels show magnified images of the upper insets. S100A8 and S100A9 proteins were detected in peri-tumoral stromal tissues (black arrowheads) and adjacent normal epithelial cells (black arrows). On right, non-tumor-bearing mouse fatpad were stained with S100A8 and S100A9. Scale bar: 100 um (**g**) Results of phospho-protein array experiments are shown. Spots with more than two-fold changes are marked with numbered rectangles and listed below (upper). The overall quantification results of phospho-protein array are shown (lower). *P < 0.05, **P < 0.01, ***P < 0.001. P values are determined by the unpaired Student t-test for d and e and Mann–Whitney test for c. C/C denotes for co-cultured MCF10A cells.

To understand the molecular mechanisms underlying the phenotype changes induced by direct co-culture, we analyzed the transcriptome profiles of MCF10A cells when they were directly co-cultured with MDA-MB-231 cells. A total of 151 genes were dysregulated in directly co-cultured MCF10A cells more than two-fold in expression (Fig. 3a, Supplementary Table S1). Again, we observed the decreased expression of mammary epithelial differentiation markers such as CDH1 or CD24^17^. Based on the gene expression profiles, we identified several cancer-related pathways, including metabolic pathway, cell adhesion, growth factor signaling, and TP53 pathway, that were significantly dysregulated in MCF10A cells after the direct co-culture (Fig. 3b).

Among the differentially expressed genes, S100A8 and S100A9 genes were highly upregulated in directly co-cultured MCF10A cells and their increased expression was also seen with qPCR and western blotting (Fig. 3c). Additionally, the mRNA dataset from Finak et al.^18^ showed that S100A8 and S100A9 genes were significantly upregulated in the human breast cancer stromal tissues (Fig. 3d). We also examined the S100A8 and S100A9 mRNA levels in normal breast and breast cancer tissues using RNA sequencing data comprised of 65 normal and 68 cancer tissues. Both genes were significantly upregulated in breast cancer tissues when compared to normal breast tissues (Fig. 3e). Interestingly, the treatment of conditioned media from MDA-MB-231 cells also resulted in up-regulation of S100A8 and S100A9 while indirect co-culture did not show such changes (Supplementary Fig. S4a).

We then used syngeneic mouse mammary carcinoma model to determine whether the S100A8/A9 expression in mammary epithelial cells can be induced by adjacent murine mammary carcinoma cells in vivo. BALB/c mouse fatpads were injected with 4T1 murine mammary carcinoma cells and were subsequently harvested. Compared to non-tumor-bearing fatpads, mouse mammary epithelial cells and peritumoral stromal cells showed increased expression of both S100A8 and S100A9 (Fig. 3f). The S100A8/9 expression and myoepithelial marker CK14 showed co-localization patterns (Supplementary Fig. S4b). These findings in addition to the above transcriptome data indicate that breast cancer cells induce S100A8/9 expression in tumor microenvironment cells including adjacent non-tumorigenic mammary epithelial cells.

To determine the changes in the cell signaling in co-cultured MCF10A cells, we used the phosphorylation antibody array to profile the phospho-protein signaling pathways. Many of the signaling pathways showed substantial dysregulation. PRAS40, STAT6, ERK1/2 levels were substantially increased while HSP60, β-catenin, RSK1/2/3, STAT3, and p53 levels were downregulated (Fig. 3g, Supplementary Fig. S4c). These results suggest that, in addition to the changes in the gene expression profiles shown above, non-tumorigenic mammary epithelial cells undergo a significant shift in signaling pathways when co-cultured with cancer cells.

### S100A8 upregulation contribute to the phenotypic and molecular changes in the co-cultured MCF10A cells

**Figure 4:**
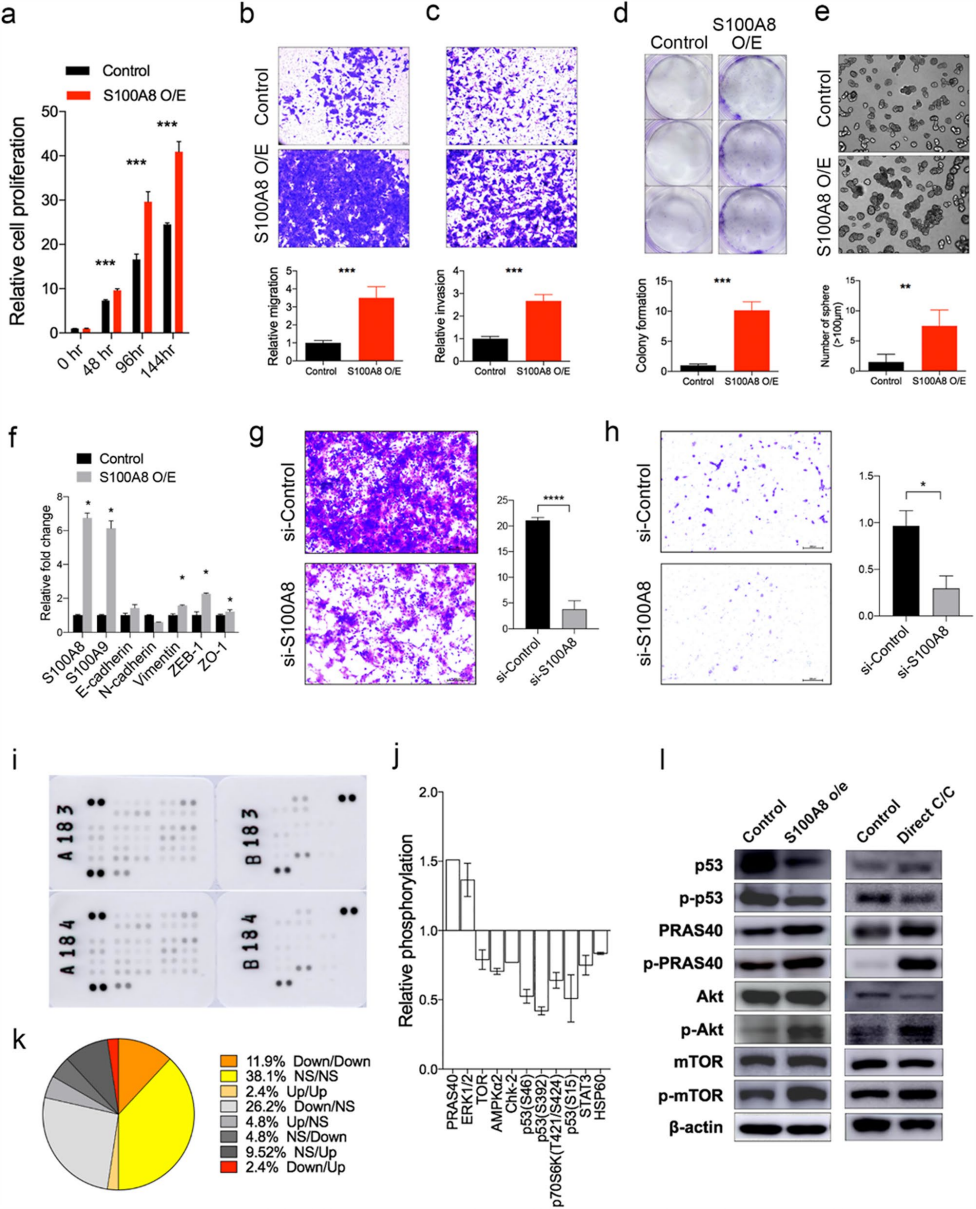
The S100A8-overexpression induce the phenotypes of non-tumorigenic mammary epithelial cells co-cultured with breast cancer cells. (**a**) Relative growth rate of S100A8/A9-overexpressing MCF10A cells was determined based on ATP level with CellTiter-Glo reagent. The representative images (upper) and quantified results (lower) of transwell migration assay (**b**), invasion assay (**c**), colony-formation assay (**d**), and the 3-dimensional matrigel culture assay (**e**) for S100A8-overexpressing MCF10A cells are shown. (**f**) The expression levels of epithelial-mesenchymal transition markers were measured by qPCR for S100A8-overexpressing MCF10A cells. The representative images (left) and quantified results (right) of transwell migration assay (**g**) and invasion assay (**h**) after S100A8 siRNA treatment in MCF10A cells are shown. (**i,j**) Results of phosphor-protein array experiments with S100A8/A9-overexpressing MCF10A cells (**i**) and quantified expression of significantly dysregulated proteins (**j**) are shown. (**k**) The comparison of phospho-protein array patterns for co-cultured MCF10A cells and S100A8/9-overexpressing MCF10A cells are shown. For example, ‘Down/Up’ designates spots that are down-regulated in co-cultured MCF10A cells and up-regulated in S100A8/A9-overexpressing MCF10A cells, respectively. (**l**) Western blotting results showing the protein expression levels of selected dysregulated proteins in both co-cultured MCF10A cells and S100A8-overexpressing MCF10A cells. Full-length blots are presented in Supplementary Fig. S6. Error bars denote mean ± SD. **P < 0.01, ***P < 0.001. P values are determined by the Mann–Whitney test.

To determine the functional importance of S100A8 and S100A9 gene expression in mammary epithelial cells, we established a stable MCF10A cells that overexpress S100A8 and S100A9 genes. Transduction of S100A8 overexpression vector resulted in upregulation of both S100A8 and S100A9 proteins in MCF10A cells (Supplementary Fig. S4d). S100A8-overexpressing MCF10A cells showed higher rate of cell proliferation when compared to the control cells (Fig. 4a). Furthermore, S100A8-overexpressing MCF10A cells showed increased cell migration, invasion, colony formation, and 3-dimensional cell growth which are characteristics of the MCF10A cells directly co-cultured with breast cancer cells. (Fig. 4b–e). Several EMT markers, such as, Vimentin and ZEB-1 showed increased expression in S100A8-overexpressing MCF10A (Fig. 4f). However, we could not observe distinct morphologic changes in S100A8-overexpressing MCF10A cells (Supplementary Fig. S4e). Additionally, we silenced S100A8 in MCF10A by using siRNA (Supplementary Fig. S4f) and observed a significant reduction in cell invasion and migration (Fig. 4g,h). Treatment of si-S100A8 resulted in a mild reduction of MCF10A cell proliferation (Supplementary Fig. S4g).

To investigated the effect of S100A8 expression on the signaling pathway activities, we performed a phosphorylation antibody array in S100A8-overexpressed MCF10A cells (Fig. 4i,j). More than half of the signaling proteins observed in S100A8-overexpressed MCF10A cells showed concordant expression patterns with directly co-cultured MCF10A cells and only one protein showed opposite expression pattern between the two cells (Fig. 4k). The dysregulation of signaling pathways in both S100A8 overexpressing MCF10A cells and the MCF10A cells directly co-cultured with MDA-MB-231 cells were further validated using western blotting as shown in Fig. 4l (Supplementary Fig. S5a). Taken together, out data demonstrate that, at least in part, the phenotypic and molecular transitions seen in the mammary epithelial cells when co-cultured with breast cancer cells can be explained by the upregulation of S100A8.

## Discussion

Normal mammary epithelial cells are closest neighboring cells to malignant epithelial cells during the initial process of carcinogenesis. Also, cancer cells often contact adjacent normal epithelial cells during the invasion process. The underlying hypothesis of our work was that normal epithelial cells adjacent to cancer cells may undergo molecular and functional transition despite the normal microscopic appearance. Indeed, we observed that non-tumorigenic epithelial cells undergo a substantial molecular and phenotypic changes when the cells were directly exposed to the breast cancer cells. Non-tumorigenic mammary epithelial MCF10A cells showed significant increase in cell proliferation and migration when they were directly co-cultured with breast cancer cells. Indirect co-culture or treatment with conditioned media resulted in minimal or moderate effect on MCF10A cells. Along with the changes in cell behavior, directly co-cultured MCF10A cells also exhibited spindle-shaped morphologies and significant downregulation of epithelial adhesion markers such as E-cadherin and Zo-1. Previous studies have shown that E-cadherin expression in normal epithelial cells plays an important role during the process initial carcinogenesis by regulating cell protrusion formation of neighboring transformed cells^12,19^. Additionally, three-dimensional direct co-culture with breast cancer cells induced loss of epithelial differentiation features in non-tumorigenic MDCK cells^20^. Finally, Trujillo et al.^21^ have shown that normal epithelial cells adjacent breast tumors show increased expression of EMT markers such as α-SMA and S100A4. Our data, along with these previous reports, suggests that breast cancer cells may induce epithelial mesenchymal transition in adjacent normal mammary epithelial cells via direct cell–cell contacts.

Using the in vitro direct co-culture approach, we observed dynamic and complex cell–cell interactions between breast cancer cells and non-tumorigenic mammary epithelial cells that include diverse physical contacts and vesicle transfers. We were also able to demonstrate that the tumor cells and normal epithelial cells exhibit various physical interactions in vivo by showing live images taken from the mouse earlobe model. Such dynamic interactions have also been reported in a three-dimensional co-culture experiment^21^ and between various stromal cell types in microenvironment^22^. Recently, Roh-Johnson et al.^23^ have shown that the cytoplasmic transfer during the cell–cell contacts between melanoma cells and macrophages is critical in cancer cell invasion in vivo. Interestingly, we observed that purified extracellular vesicles obtained from breast cancer cells exert a pro-proliferative effect in MCF10A cells similar to those seen in the direct co-culture. The molecular mechanism of this extracellular vesicle-induced effect on normal epithelial cells should be investigated in future researches.

The clinical and biologic consequences of this cell–cell interactions between malignant cancer cells and adjacent normal epithelial cells are unclear. Normal epithelial cells exert tumor-suppressive effects during the initial phase of carcinogenesis by cell competition and protrusion of neighboring transformed cells^19,24,25^. Alternatively, tumor cells can reprogram the adjacent normal epithelial cells to form a pro-tumorigenic microenvironment at the late stages of carcinogenesis^13^. This phenomenon of tumor-driven reprograming of microenvironment has been shown for other cell types such as fibroblast or adipocytes in breast cancer^5,6^. Further research is needed to clarify the role of molecular transformation of normal epithelial cells during the breast cancer progression.

In molecular levels, we observed that S100A8/A9 expression levels are significantly up-regulated when non-tumorigenic MCF10A epithelial cells were co-cultured with MDA-MB-231 cells. The up-regulation of S100A8/A9 in mammary myoepithelial cells adjacent to the cancer cells was also observed in the 4T1 syngeneic mouse model tumors. S100A8/A9 act as alarmins that trigger damage-associated molecular patterns molecules and modulate the immune response^26^. S100A8/A9 overexpression in MCF10A cells resulted in a similar cell phenotype seen in directly co-cultured MCF10A cells in terms of in vitro cell behaviors and cell signaling activities. Moon and colleagues have previously shown that S100A8/A9 overexpression can induce an invasive signaling program in breast epithelial cells via the H-RAS activity^27,28^. Our results show that S100A8/A9, potentially critical regulators of cell behavior, could be induced in non-tumorigenic mammary epithelial cells during the dynamic cell–cell interactions with adjacent breast cancer cells. Additionally, the directly co-cultured and S100A8/A9-overexpressing cells both showed increased phosphorylation of PRAS40-Thr^246^ and its upstream Akt-Ser^473^. Phosphorylation of Akt-Ser^473^ and PRAS40-Thr^246^ activate mTOR signaling that affects diverse biologic aspects of the epithelial cells and other cells of tumor microenvironment^29–31^. The biologic implications and therapeutic potentials of the observed mTOR signaling dysregulation in mammary epithelial cells in breast cancer microenvironment should be further explored.

Our study has several limitations. First, we have used a limited number of cell lines during the present study. The molecular transitions in non-tumorigenic mammary epithelial cells might vary along the different subtypes of breast cancers. Second, the effect of this phenotype changes in mammary epithelial cells on the breast cancer cells in terms of breast cancer growth and metastasis has not been investigated using in vivo breast cancer models. Also, we cannot rule out the possibility that the varying degrees of the phenotype changes observed in the MCF10A cells under different co-culture methods are caused by the different concentration of secreted materials from breast cancer cells since we could not experimentally adjust for the concentrations for each method. Additionally, the upregulation of S100A8/9 in the breast cancer stromal tissues should be further examined by using a large dataset of triple negative breast cancer patients. Although we have observed increased S100A8/9 expression in a publicly available gene expression dataset^18^, the dataset does not provide additional information on tumor subtypes. Although we’ve demonstrated the up-regulation of S100A8/A9 expression in mouse fat pad xenograft tumors, we were not able to examine the S100A8/A9 up-regulation in human breast cancer tissues in the present study. Further studies investigating the biologic consequence and clinical implications of the molecular changes in the non-tumorigenic mammary epithelial cells in breast cancer microenvironment are needed. Finally, the biologic significance and the underlying molecular mechanism of the direct cell–cell interaction using lamellopodia or nanotubes is currently unclear.

In conclusion, our study demonstrate that breast cancer cells may induce substantial molecular changes in non-tumorigenic mammary epithelial cells via dynamic cell–cell interactions. As the results, mammary epithelial cells undergo a phenotype transition which involves more active proliferation and migration. S100A8/A9 may play a pivotal role during this phenotype transition of mammary epithelial cells. Our study provides scientific basis for pursuing a novel therapeutic strategy that targets the non-tumorigenic mammary epithelial cells in tumor microenvironments.

## Methods

### Cell culture

Cells were purchased from KOREAN CELL LINE BANK (Seoul, Korea). Non-tumorigenic mammary epithelial MCF10A cells were maintained in a 1:1 mixture of Dulbecco’s Modified Eagle’s Medium (DMEM) and Ham's F12 medium (F12) with 5% horse serum, 20 ng/mL epidermal growth factor (EGF), 100 ng/mL cholera toxin, 10 μg/mL insulin, and 500 ng/mL hydrocortisone. MCF7 and MDA-MB-231 breast cancer cells were cultured in DMEM with 10% FBS, 1% penicillin, and 1% streptomycin. SK-BR3 breast cancer cells were cultured in RPMI 1640 with 10% FBS, 1% penicillin, and 1% streptomycin. For fluorescence tagged cells, puromycin was added.

### Co-culture of MCF10A cells and breast cancer cells

To optimize the culture medium for co-culture, we tested the effect of various mixture of MDA-MB-231 culture media and MCF10A culture media on cell survival. For effective separation of MCF10A cells and MDA-MB-231 cells after the direct co-culture, MCF10A cells and MDA-MB-231 cells were transfected with GFP and RFP using lentiviral vectors, respectively. After the direct co-culture, cells were isolated using a FACS Aria II cell sorter (BECKTON DICKINS, NJ) as shown in the Supplementary Fig. S2. A total of 1.0 × 10^6^ mixture of cells were used for the direct co-culture with different ratio (3 × 10^5^ MCF10A and 7 × 10^5^ MDA-MB-231 for 3:7 ratio and 7 × 10^5^ MCF10A and 3 × 10^5^ MDA-MB-231 for 7:3 ratio) in 150 mm^3^ dish during 96 h. After sorting, the GFP-labeled MCF10A cells were stabilized with MCF10A media for 24 h and then used for various assays. Based on the effect on cell proliferation, we chose 7:3 ratio mixture of MDA-MB-231 media and MCF10A media for the direct co-culture of the cells (Supplementary Fig. S1c).

Indirect co-culture was performed by using the transwell insert (pore size 0.4 μm). 2 × 10^4^ MDA-MB-231 cells (experimental) or 2 × 10^4^ MCF10A cells (control) were seeded on the membrane of the insert (pore size 0.4 μm) with serum free basal media. 5 × 10^4^ MCF10A cells were seeded in the lower 24 well plates with 500 μL FBS added to the MCF10A media. The cells were cultured for three days and then used for various assays.

### The preparation of MDA-MB-231 conditioned media

MCF10A cells were cultured by aforementioned method. 2 × 10^6^ MDA-MB-231 cells were cultured in 100 mm^3^ dishes with 10 mL 10% FBS-DMEM. After 24 h, cells were washed with PBS and were cultured with 5 mL serum free Ham's F12 medium and 5 mL serum free DMEM for 24 h. Then, 10 mL of the supernatant was collected and stored at − 20 °C. We did not use any concentration protocols for the conditioned media.

### In vitro assays measuring cell phenotypes

Proliferation assays were conducted using CellTiter-Glo Luminescent Cell Viability Assay (PROMEGA, Madison, USA) following the manufacturer’s protocol. Cells were seeded in triplicate into 96-well plates at a density of 2,000 cells per well. For migration assay, 2 × 10^4^ cells were seeded in an insert (8 μm pore size) with serum free media and media with 10% FBS was added in lower chambers. Cells were incubated for 20 h and fixed with 4% paraformaldehyde and stained with 0.1% crystal violet. For colony formation assay, 2 × 10^3^ cells were seeded into 6-well plates. After 2 weeks, colonies were fixed in 4% paraformaldehyde and stained with 0.1% crystal violet.

### RNA sequencing and qPCR

RNA sequencing libraries were prepared using TruSeq RNA Access library kit (Illumina, Inc., San Diego, CA, USA) according to the manufacturer`s protocol. After validation of the libraries, using Agilent DNA screentape D1000 kit on a TapeStation (Agilent Technologies, Santa Clara, CA, USA), the hybridization steps were performed using exome capture probes and streptavidin coated beads. RNA sequencing was performed by HiSeq 2000 (Illumina,San Diego, USA by the Macrogen Incorporated). We processed reads from the sequencer and aligned them to the Homo sapiens (hg19). After aligning reads to genome, Cufflinks v2.2.1 was used to assemble aligned reads into transcripts and to estimate their abundance. The transcript counts in isoform and gene level were calculated, and the relative transcript abundances were measured in FPKM (Fragments Per Kilobase of exon per Million fragments mapped) from Cufflinks.

RNA sequencing data of breast cancer was generated through a separate project to characterize the genomic profiles of the primary breast tumor and patient-derived xenograft tumors which will be presented in other reports (IRB No. 1402-054-555).

For qPCR, total RNA was extracted from isolated cells with TRIzol (FAVORGEN, Taiwan). PrimeScript 1st strand cDNA Synthesis Kit (TAKARA, Japan) were used for reverse transcription of RNA, and resulting cDNA was amplified using Power SYBR Green PCR Master Mix (APPLIED BIOSYSTEM, CA). The information on the primer sequences used in this study is listed in the Supplementary Table S2.

### Phosphorylation array and western blotting

For phosphorylation array, protein extraction was done with buffer following the manufacturer’s protocol. Protein concentration was measured by BCA assay kit (THERMO SCIENTIFIC, Palm Springs, CA, USA). Phosphorylation array was performed by using the human phosphor-kinase array kit (R&D SYSTEMS, USA).

Proteins were harvested with RIPA buffer (THERMO SCIENTIFIC, Palm Springs, CA, USA), protease & phosphatase inhibitor and 0.5 M EDTA solution. Protein concentration was measured by BCA assay kit (THERMO SCIENTIFIC, Palm Springs, CA, USA). Cell lysates were loaded onto 10% gels and transferred to a PVDF membrane. The membrane was blocked with 5% skim milk and incubated with primary antibody overnight at 4℃. The peroxidase-conjugated secondary antibody was used for detection. Bands were detected by LAS. The information on the antibodies used in this study is listed in the Supplementary Table S3.

### Cell imaging

For time-lapse imaging of cells during the direct co-culture, 9 × 10^3^ GFP-transfected MCF10A cells and 2 × 10^4^ RFP-transfected MDA-MB-231 cells were seeded in 8-well chambers and cultured for 24 h. After 24 h culture, live cells images were taken every 10 min with fixed position for 16 h by using Leica confocal microscope (Leica TCS SP8, LEICA MICROSYSTEMS, Ltd, Korea), and the obtained images were analyzed by Leica LAS X program.

For in vivo imaging, the experiments were performed in accordance with the Animal Care and Use Committee guidelines of WOOSUNG BSC (Suwon, Korea). Hair-removed ear skin of C57BL/6 mouse (10 weeks old) was injected with 4.6 × 10^4^ cells of the mixture MCF10A-GFP (2.3 × 10^4^) and MDA-MB-231-RFP (2.3 × 10^4^) cell lines. The mice were anesthetized with an intraperitoneal injection of Zoletil (30 mg/kg, VIRBAC, Carros, France) and Rompun (10 mg/kg, BAYER-KOREA, Seoul, Korea) before imaging. The mice were placed on the heated plate of a motorized XYZ translational stage. The in vivo movement of MCF10A and MDA-MB-231 was monitored by modified custom-built laser-scanning confocal microscopy {Choe, 2015 #3}. GFP-expressing cells were visualized at an excitation wavelength of 491 nm and detected through a bandpass filter of 502 nm to 537 nm (SEMROCK Inc, Rochester, NY, USA). RFP-expressing cells were imaged at an excitation wavelength of 532 nm and detected using a bandpass filter of 562 nm to 596 nm (SEMROCK Inc). Cell movement was visualized at 1 min interval for 2 h. After acquired from the imaging system, 512 × 512 pixel images were then compensated with Matlab (MATHWORKS, Natick, MA, USA) and reconstructed by ImageJ software.

### S100A8/A9 and CK14 expression in xenograft tumor-bearing mouse fatpad

5-week-old BALB/c mice were purchased from KOATECH (Seoul, Korea) and housed in Seoul National University Hospital Clinical Research Institute's Specific Pathogen Free zone. All experiments were approved by the Institutional Animal Care and Use Committee in Seoul National University Hospital (SNUH-IACUC, 17-0165-S1A0 (1)) and done in accordance with the ARRIVE guideline. To obtain control mouse fatpads, eight-week-old non-tumor-bearing BALB/c mouse was sacrificed and mammary fat pad was resected. To obtain tumor-bearing mouse fat pad, 2 × 10^5^ 4T1 mouse mammary epithelial cancer cells were injected in 6-week-old BALB/c mouse's mammary fat pad. At two weeks after the tumor injection, the mouse was sacrificed and tumor-bearing mammary fatpad was resected. The resected fatpads were fixed with 4% PFA, embedded in paraffin, and used for the immunohistochemistry against S100A8/A9 and CK14.

### Establishment of the S100A8/A9-overexpressing MCF10A cell line and S100A8 siRNA treatment

The coding sequences of S100A8 and S100A9 were acquired by RT-PCR and cloned into pCDH-GFP and pCDH-RFP vectors. MCF10A cells were transfected with lentivirus S100A8-GFP and S100A9-RFP construct. Transfected cells were selected by puromycin. For S100A8 silencing in MCF10A cell line, we used S100A8 siRNA product (BIONEER, Korea). Twenty-four hours after seeding the cells, we treated MCF10A cells with 25 pmol siRNA diluted in Lipofectamine RNAi MAX reagent (INVITROGEN, USA) for 48 h.

### Phalloidin staining

We performed phalloidin staining to quantify the degree of morphological changes in MCF10A cells. we fixed the cells with 4% paraformaldehyde in PBS at room temperature for 20 min. After washing the cells, we added 0.1% triton X-100 in PBS for 5 min to increase permeability. The cells were stained by Alexa-488 conjugated phalloidin for 90 min followed by DAPI staining. After washing cells with PBS, we observed the cells with fluorescence microscope at Ex/Em 493/517 nm.

### Preparation and quantification of the extracellular vesicles

We used Exo-spin (CELL GUIDANCE SYSTEM, MO, USA) to purify the extracellular vesicles from breast cancer cells. Briefly, we cultured 1 × 10^6^ MDA-MB-231 using 150-cm dish for 3 days in DMEM and exosome-free FBS. After removing remaining cells and cell debris in the culture medium by centrifugation (300×*g*, 10 min, and 16,000×*g*, 30 min), the supernatant was transferred to a new centrifuge tube and 50% volume of Exo-spin buffer was added for overnight incubation. After additional centrifugation (16,000×*g*, 1 h), we obtained exosome-containing pellet that was eluted in PBS. We used ExoView kit (NANOVIEW BIOSCIENCE, MA, USA) and ExoView R100 Instrument to quantify the amount of extracellular vesicles.

### Statistical analysis

In general, most data represent the mean ± S.D and are representative of 3 independent experiments, except for RNA sequencing and phospho-protein arrays which are two independent experiments. Graph Pad Prism (ver. 7.01) was used for generating graphs and heatmaps and performing statistical tests. P values were calculated from unpaired two-tailed Student’s t tests or Mann–Whitney test as appropriate.

To identify differentially expressed genes, we filtered genes with one more than zeroed FPKM values and the data were log2-transformed and subjected to quantile normalization. Statistical significance of the differential expression data was determined using fold change in which the null hypothesis was that no difference exists among samples. Gene pathway analysis for the DEG was done based on KEGG pathway (http://www.genome.jp/kegg/pathway.html).

## Supplementary Information

Supplementary Tables.

Supplementary Video 1.

Supplementary Video 2.

Supplementary Video 3.

Supplementary Video 4.

Supplementary Information 1.

## Data Availability

All data supporting our findings can be found in the main paper or in supplementary files.
