# Supplementary material for: S100A8/A9 mediate the reprograming of normal mammary epithelial cells induced by dynamic cell–cell interactions with adjacent breast cancer cells
Source: Sci Rep. 2021 Jan 14;11:1337. doi: 10.1038/s41598-020-80625-2 (PMC7809201; doi:10.1038/s41598-020-80625-2)
Supplement: Supplementary file 6 — Supplementary Information 1. [file 41598_2020_80625_MOESM6_ESM.docx]

**Title: S100A8/A9 mediate the reprograming of normal mammary epithelial cells induced by dynamic cell-cell interactions with adjacent breast cancer cells.**

Running title: Cancers induce phenotypic changes in normal cells

Seol Hwa Jo^1*^, Woo Hang Heo^1*^, Hye-Youn Son^2*^, Mingji Quan^1^, Bok Sil Hong^2^, Ju Hee Kim^2^, Han-Byoel Lee^3^, Wonshik Han^2,3,4^, Yeonju Park^5^, Dong-Sup Lee^5^, Nam Hoon Kwon^6^, Min Chul Park^6^, Jeesoo Chae^4,7^, Jong-Il Kim^4,7^, Dong-Young Noh^3,4,8^, Hyeong-Gon Moon ^2,3,4^.

^1^Interdisciplinary Graduate Program in Cancer Biology, Seoul National University College of Medicine, Seoul, Korea,

^2^Center for Medical Innovation, Biomedical Research Institute, Seoul National University Hospital, Seoul, Korea,

^3^Department of Surgery, Seoul National University College of Medicine, Seoul, Korea,

^4^Genomic Medicine Institute, Medical Research Center, Seoul National University College of Medicine, Seoul, Korea,

**Supplementary information**

Supplementary Figure S1-8.

Supplementary Figure S2.

Supplementary Figure S3.

Supplementary Figure S4.

Supplementary Figure S5.

Supplementary Figure S6.

Supplementary Figure S7.

Supplementary Figure S8.

Supplementary Table S2-3.

Supplementary Table S1 and Supplementary Video S1-4 are not included in this Supplementary Material File. The legends for Supplementary Table S1 and Supplementary Video S1-4 are included here.

**Supplementary figure legend**


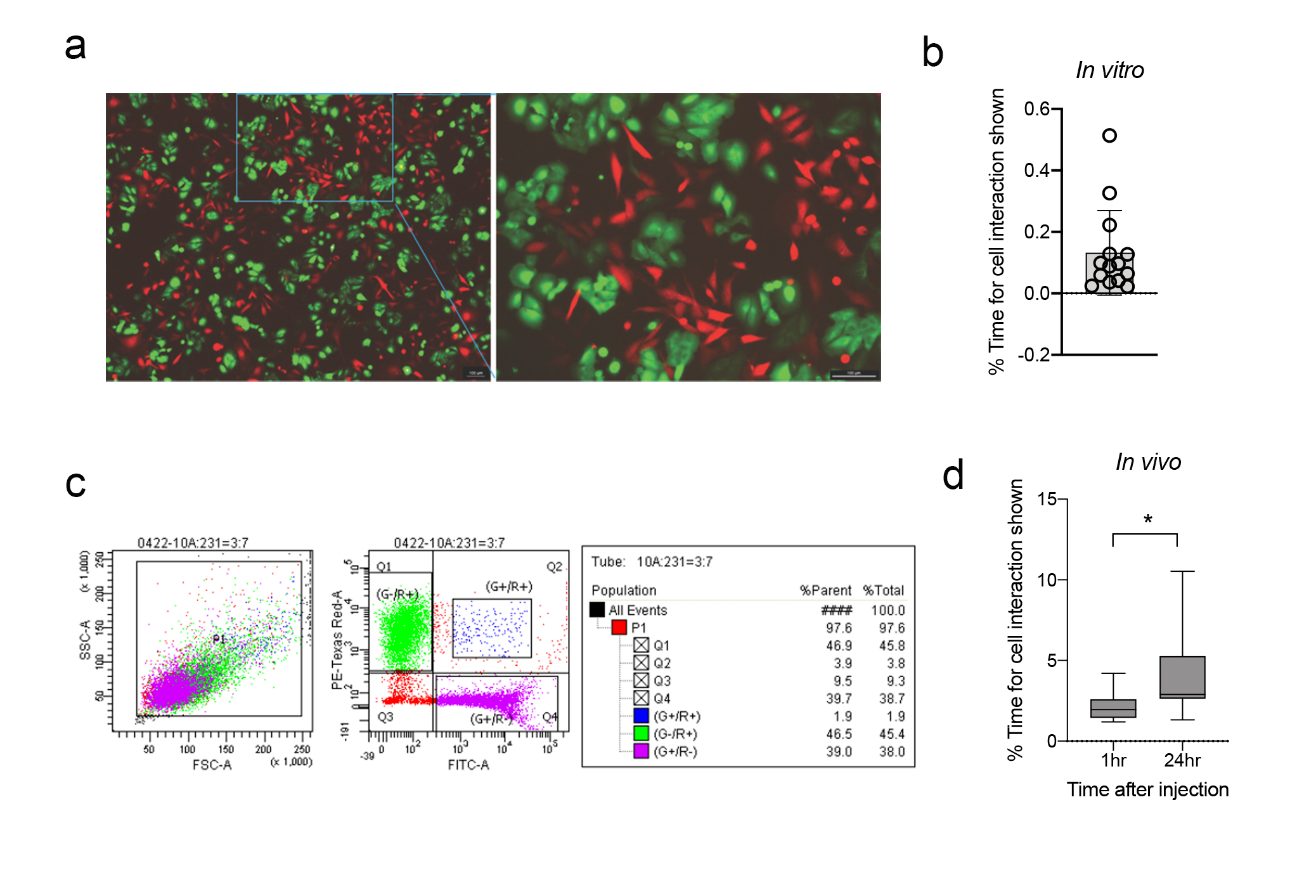


**Supplementary Figure S1. Cell-cell interaction between MCF10A and MDA-MB-231 cells *in vitro* and *in vivo*.**

**a**. Images of the MCF10A (green) and MDA-MB-231 (red) cells during the *in vitro* direct co-culture are shown. **b**. The frequency of microscopically detectable direct cell-cell interaction between MCF10A and MDA-MB-231 cells. **c**. MDA-MB-231 cancer cell (red) and MCF10A (green) were co-cultured for 3 days and then they were separated by FACS sorter. The ratio of MDA-MB-231 cancer cells: MCF10A cells was 7:3. **d**. The frequency of microscopically detectable direct *in vivo* cell-cell interaction between the MCF10A (green) and MDA-MB-231 (red) at 1hr and 24 hours after the co-injection in mouse earlobe.

**
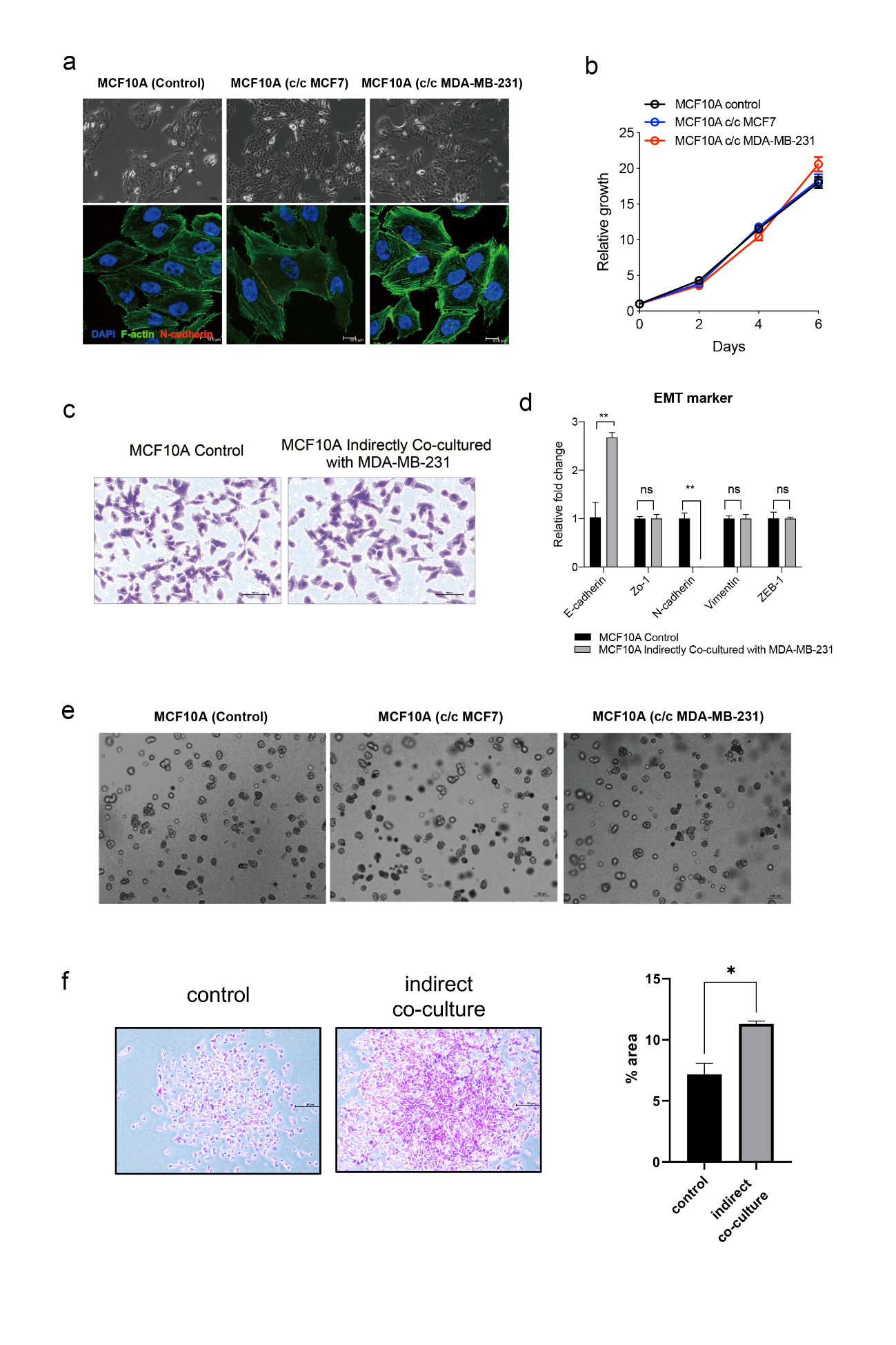
**

**Supplementary Figure S2. The effect of in-direct co-culture effect on MCF10A cells.**

**a**. Light microscopic images (upper) and F-actin staining (lower) to present cellular morphology of MCF10A cells after the direct co-culture with MCF7 and MDA-MB-231 breast cancer cells. **b**. Relative growth rate of indirectly co-cultured MCF10A cells was measured based on ATP level with CellTiter-Glo reagent. The representative images of transwell migration assay (**c**) and the mRNA expression level of epithelial-mesenchymal transition markers (**d**) in MCF10A after in-direct co-culture with MDA-MB-231. **e**. The representative images of the three-dimensional matrigel culture assay for MCF10A cells after the in-direct co-culture with MCF7 or MDA-MB-231. **f**. The representative image (left) and the quantification results (right) of colony forming assay.


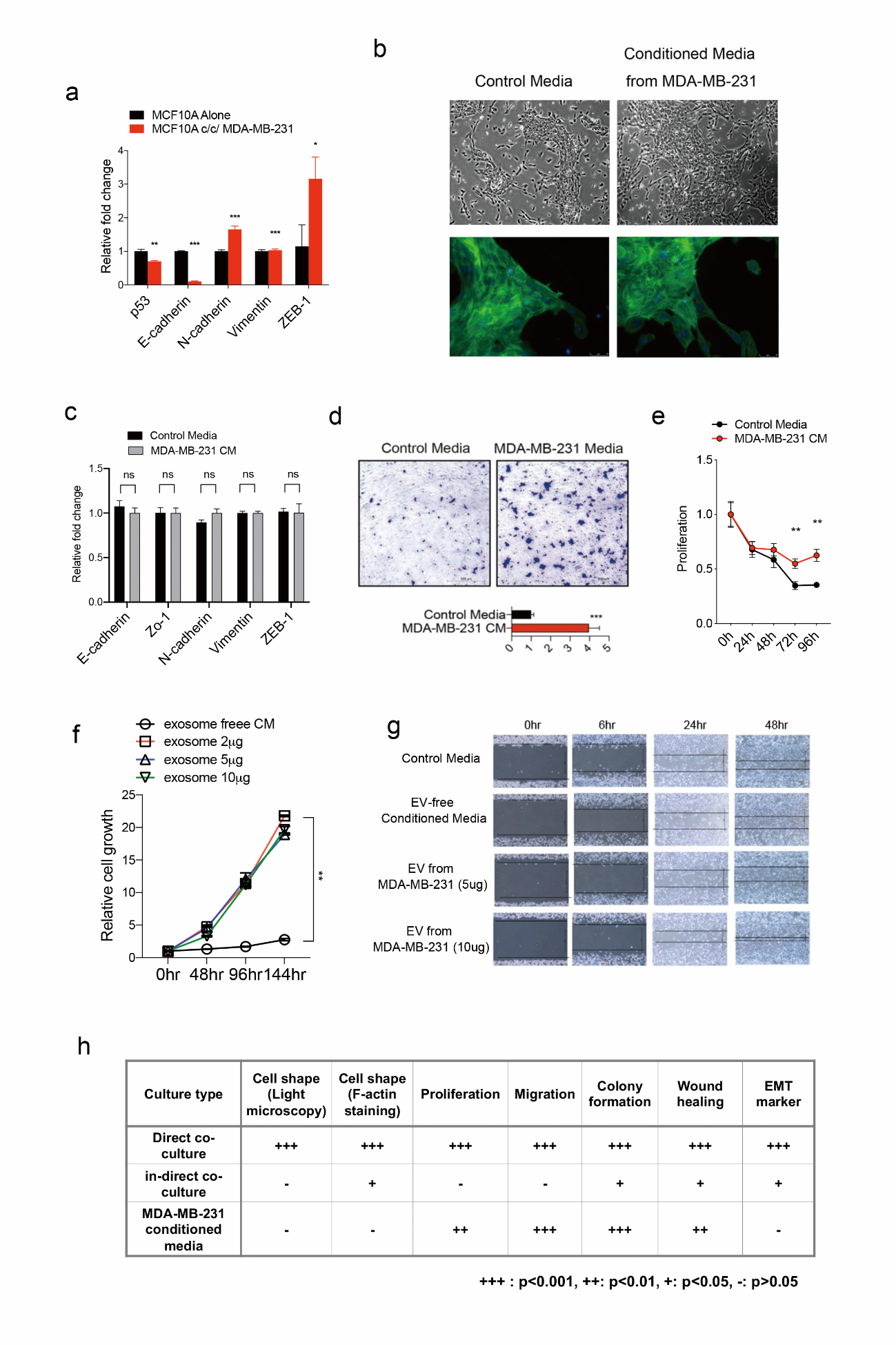


**Supplementary Figure S3. The effect of conditioned media and extracellular vesicles from MDA-MB-231 cells on MCF10A cells**

**a**. The expression levels of epithelial-mesenchymal transition (EMT) markers measured by qPCR in MCF10A cells after the direct co-culture with MDA-MB-231 cells. **b**. Cell morphology of MCF10A after MDA-MB-231 conditioned media treatment (light microscopic feature (upper) and F-actin staining (lower)). **c**. The EMT marker expression in MCF10A cells after MDA-MB-231 cell conditioned media treatment measured by qPCR. **d**. The representative images (upper) and quantified results (lower) of transwell migration assay. **e**. MCF10A cell growth under serum starvation with or without the conditioned media from MDA-MB-231 cells. Relative proliferation (**f**) and wound healing assay (**g**) after treatment of purified extracellular vesicles obtained from MDA-MB-231 cells. **h**. The table summarizes the results of various in vitro assays comparing the effect of the direct co-culture, in-direct co-culture, and conditioned media treatment on MCF10A cells.


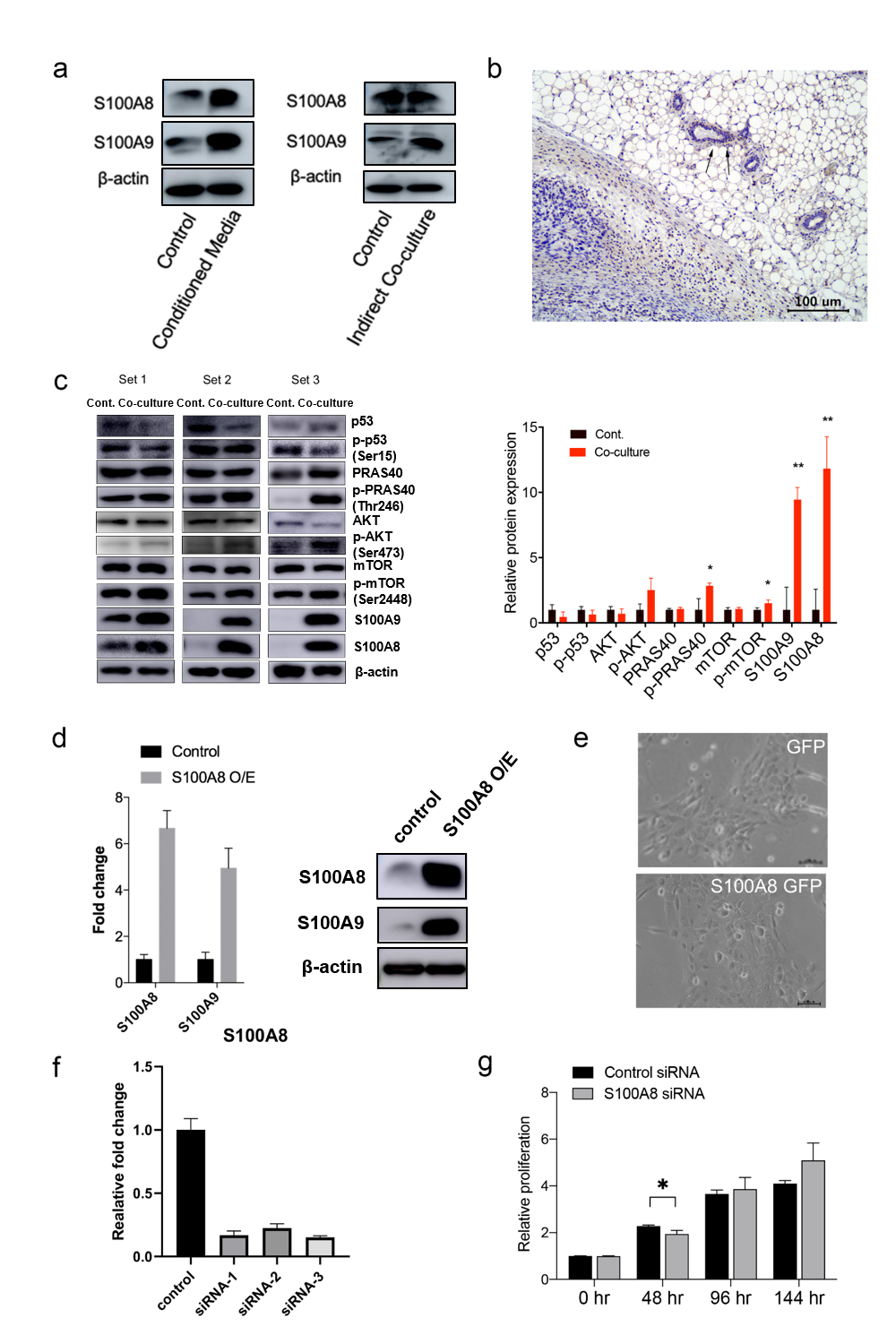


**Supplementary Figure S4. S100A8 expression and MCF10A cell phenotypes.**

**a**. The induction of S100A8/A9 gene expression in MCF10A cells after the conditioned media treatment (left) and in-direct co-culture (right). Full-length blots are presented in Supplementary Figure S6. **b**. The myoepithelial marker CK14 staining on 4T1 tumor bearing mouse mammary fat pad. **c**. Western blot validation experiment showing the changes of phosphor-proteins identified by the protein array. The triplicate raw data (left) and quantification results (right) are shown. Triplicate full-length blots are presented in Supplementary Figure S7. **d**. S100A8 and S100A9 mRNA (right) and the protein (left) in S100A8 over-expressing MCF10A cells. Full-length blots are presented in Supplementary Figure S6. **e**. Cell morphological change in S100A8 over-expressing MCF10A cells. **f**. SiRNA-mediated gene silencing of S100A8 gene mRNA measured by qPCR. **g**. MCF10A cell proliferation after siS100A8 treatment determined based on ATP level with CellTiter-Glo reagent.


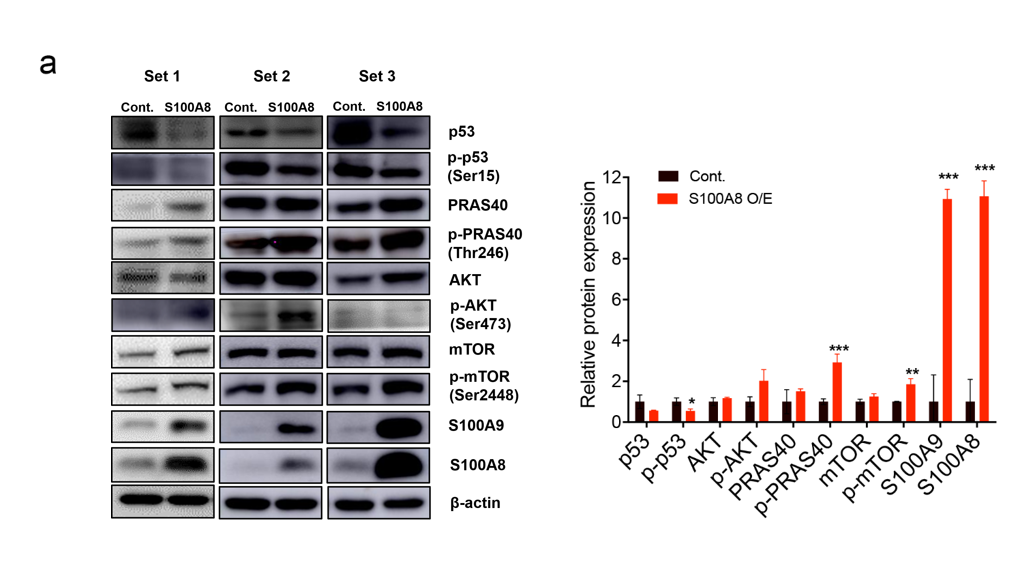


**Supplementary Figure S5. S100A overexpression and signaling pathways.**

**a.** Western blot validation experiment showing the changes of phosphor-proteins in S100A8-overexpressing MCF10A cells. The triplicate raw data (left) and quantification results (right) are shown. Triplicate full-length blots are presented in Supplementary Figure S8.

**
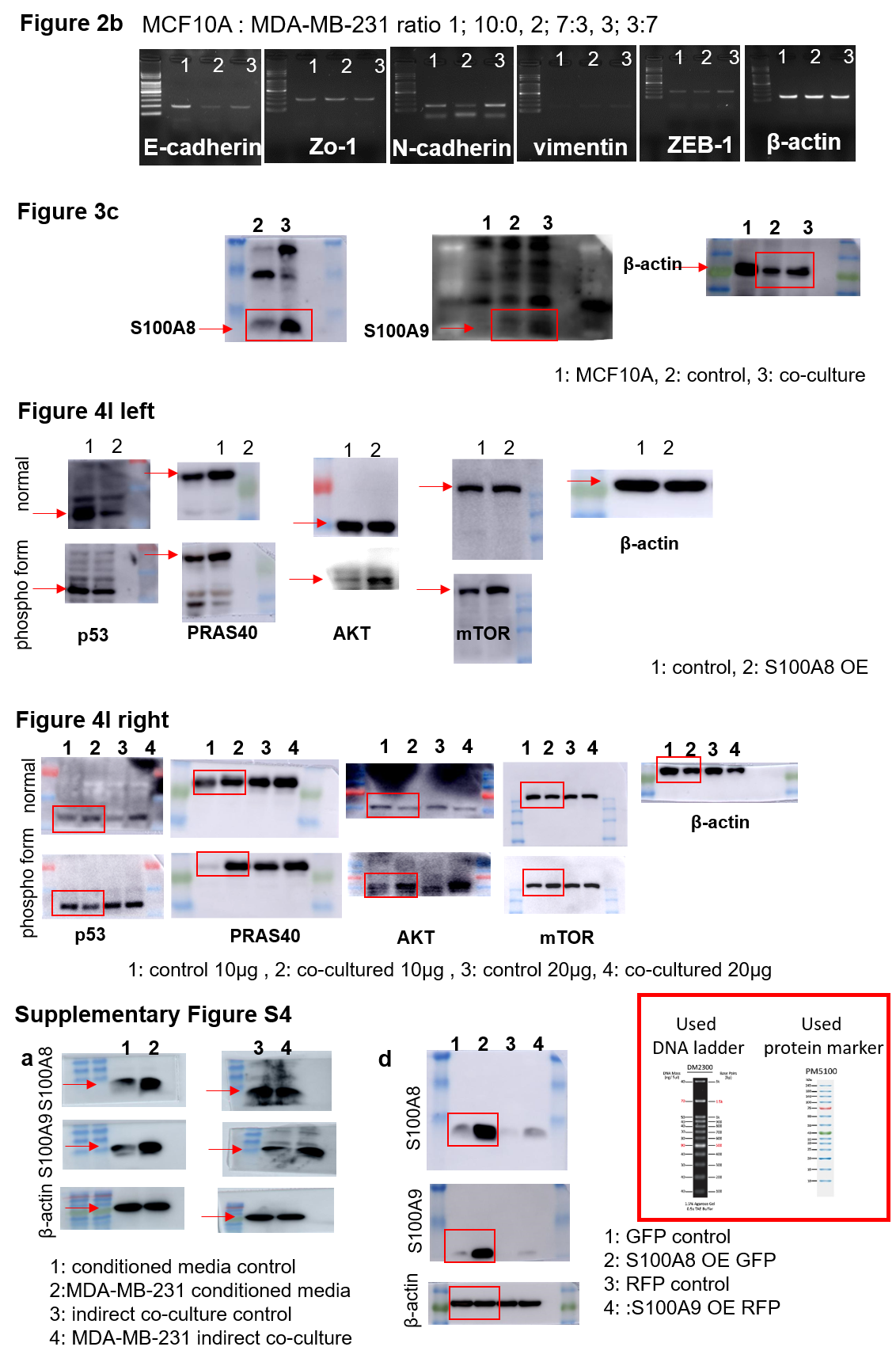
**

**Supplementary Figure S6. Full-length blots/gels**

RT-PCR full length original gel pictures of Figure 2b in the top are shown. Next, the blots of Figure 3c S100A8/A9 protein levels in MCF10A cells co-cultured with MDA-MB-231 cells are shown, but we could not find the original blot of loading control like as β-actin of S100A8 blot. In the middle of figure, Figure 4l’s original blots are shown. In the bottom of this figure, Supplementary Figure S4a and S4d blots’ are shown. Used marker and ladder are included in the edge of this figure.

**
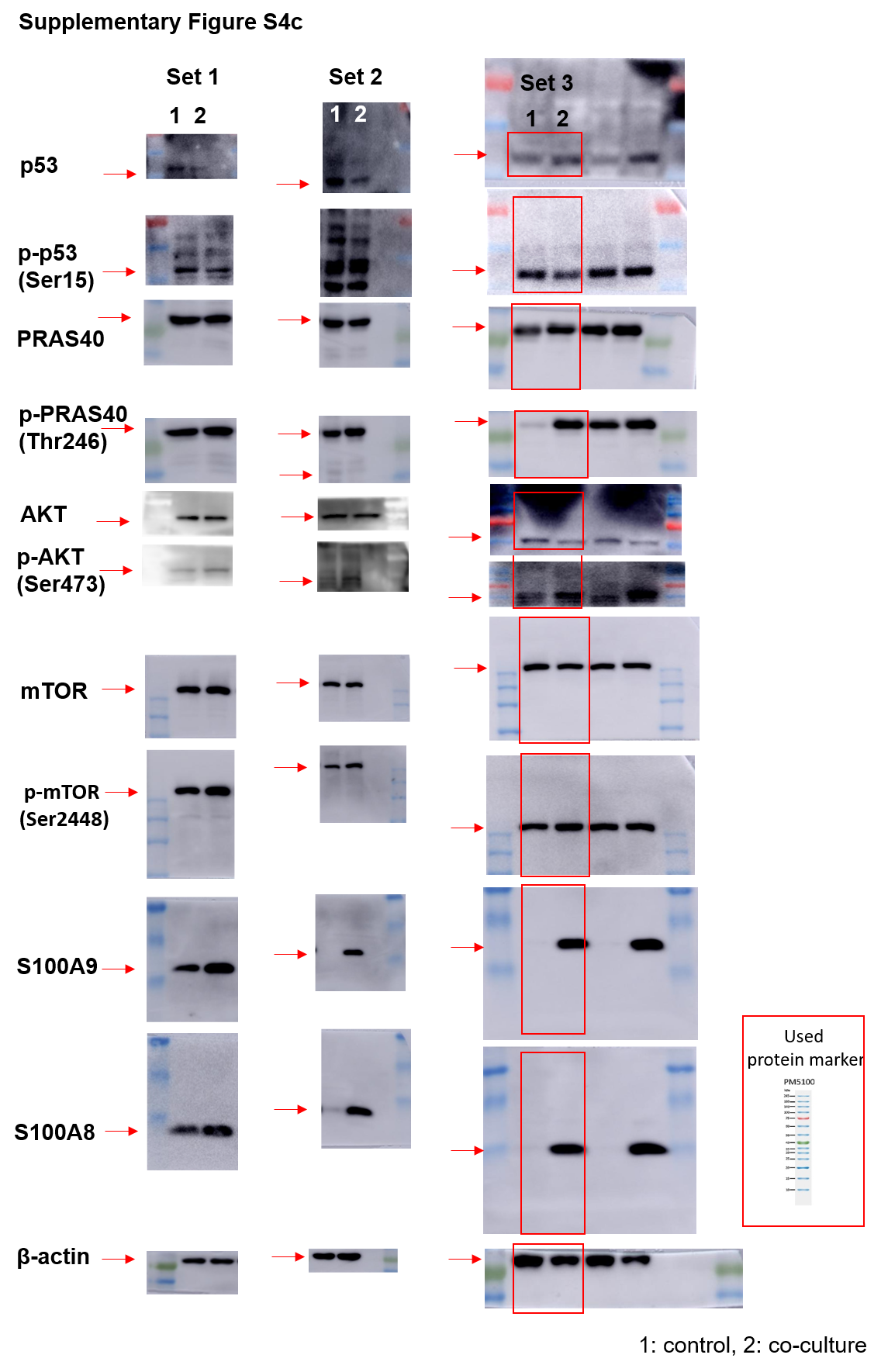
**

**Supplementary Figure S7. Full-length blots of Supplementary Figure S4c**

Full-length triplicate original blots of Supplementary Figure S4c western blot from MCF10A direct co-cultured with MDA-MB-231. 1: control, 2: co-cultured. In the right bottom, used marker are shown.

**
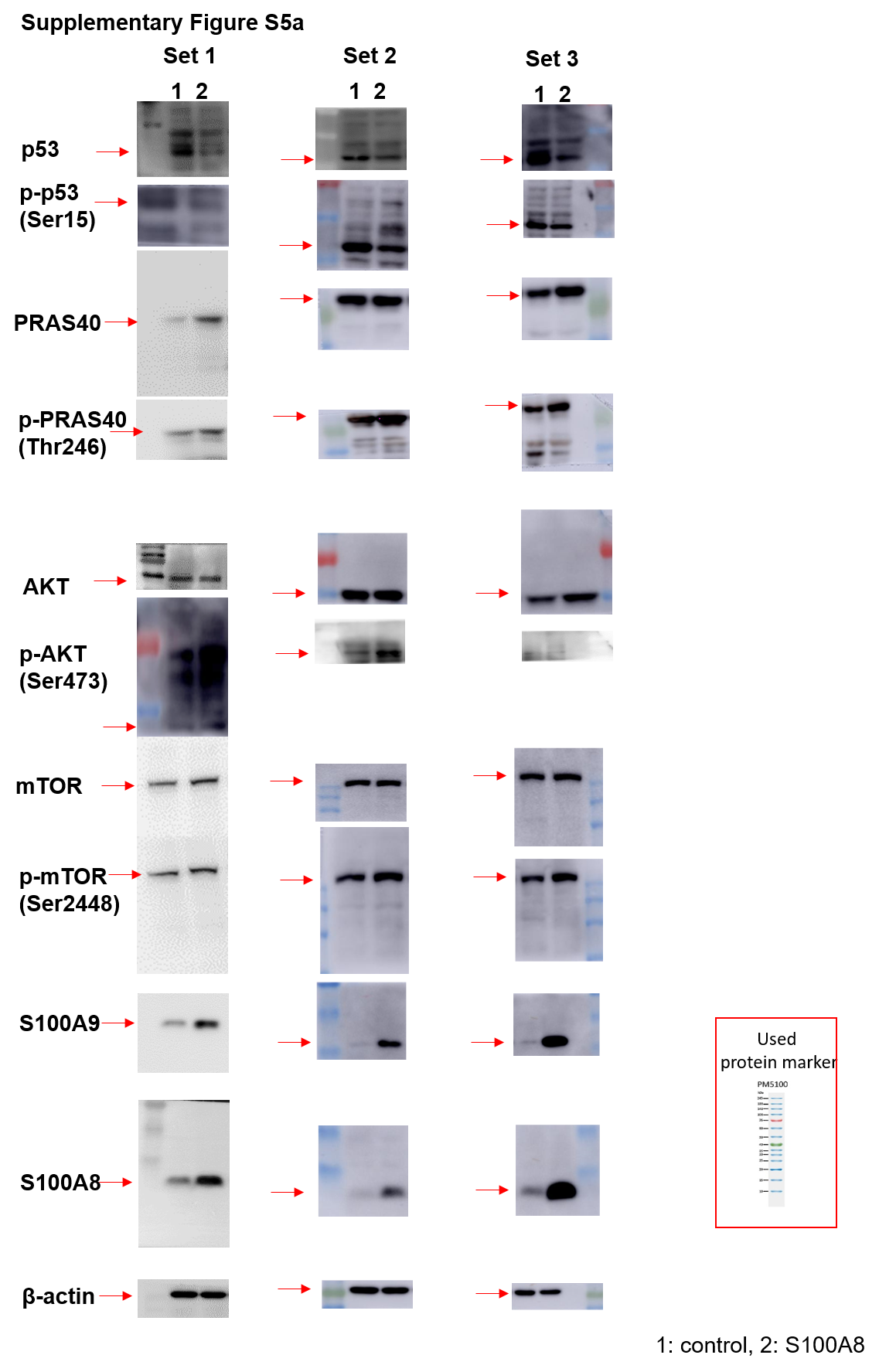
**

**Supplementary Figure S8. Full-length blots Supplementary Figure S5a**

Full-length triplicate original blots of Supplementary Figure S5a western blot from S100A8 over expressed MCF10A. 1: control, 2: S100A8 over expressed. In the right bottom, used marker are shown.

**Supplementary Table legend**

**Supplementary Table S1**. Gene expression level in MCF10A cells after the direct co-culture with MCF10A (control) and MDA-MB-231 (experimental) (Figure 3b)

**Supplementary Table S2**. Information of primers used in this study.

| Name |  | Sequences |
| --- | --- | --- |
| S100A8 | F | ATGCCGTCTACAGGGATGAC |
|  | R | CCACGCCCATCTTTATCACC |
| S100A9 | F | GGGAATTCAAAGAGCTGGTGC |
|  | R | AGCTGCTTGTCTGCATTTGTG |
| KRT17 | F | CAGTCCCAGCTCAGCATGAA |
|  | R | CCACAATGGTACGCACCTGA |
| KRT16P3 | F | TCAGACCGGTGGAGAAGTGA |
|  | R | TGTGCCGGGTCCTTCATACT |
| KRT16 | F | AGTCCCAGCTCAGCATGAAA |
|  | R | GCGGGAAGAATAGGATTGGC |
| KRT10 | F | CAACTCACATCAGGGGGAGC |
|  | R | CAGCTCATCCAGCACCCTAC |
| CD24 | F | GCTCCTACCCACGCAGATTT |
|  | R | GAGACCACGAAGAGACTGGC |
| ZEB1 | F | GTGACGCAGTCTGGGTGTAA |
|  | R | TGAGTCCTGTTCTTGGTCGC |
| Snail | F | GAGGACAGTGGGAAAGGCTC |
|  | R | TGGTTCGGATGTGCATCTT |
| ZO-1 | F | GACAGCAGACCACGTTACGA |
|  | R | TGAAGGTATCAGCGGAGGGA |
| β-actin | F | CACTGTGTTGGCGTACAGGT |
|  | R | TCATCACCATTGGCAATGAG |
| E-cadherin | F | CCCTCGACACCCGATTCAAA |
|  | R | TGGATTCCAGAAACGGAGGC |

**Supplementary Table S3**. Information of antibodies used in this study.

| Antibody | Source | Company (catalog) |
| --- | --- | --- |
| Calgranulin A | Mouse monoclonal antibody | Santa Cruz (sc-48352) |
| Calgranulin B | Mouse monoclonal antibody | Santa Cruz (sc-376772) |
| AKT | Rabbit polyclonal antibody | Cell signaling (9272) |
| p-AKT (Ser473) | Rabbit polyclonal antibody | Cell signaling (9271) |
| p53 | Mouse monoclonal antibody | Cell signaling (2524) |
| p-p53 (Ser15) | Rabbit polyclonal antibody | Cell signaling (9284) |
| PRAS40 | Rabbit monoclonal antibody | Cell signaling (2691) |
| p-PRAS40 (Thr246) | Rabbit monoclonal antibody | Cell signaling (2997) |
| mTOR | Rabbit monoclonal antibody | Cell signaling (2983) |
| p-mTOR (Ser2448) | Rabbit monoclonal antibody | Cell signaling (5536) |
| RAGE | Rabbit polyclonal antibody | AbCam (ab3611) |
| TLR4 | Mouse monoclonal antibody | Santa Cruz (sc-293072) |
| S100A8 | Mouse monoclonal antibody | Santa Cruz (sc-48352) |
| S100A9 | Mouse monoclonal antibody | Santa Cruz (sc-376772) |
| CK14 | Rabbit polyclonal antibody | BioLegend (PRB-155P) |
| β-actin | Mouse monoclonal antibody | Santa Cruz (sc-47778) |

**Supplementary video legend**

**Supplementary Video S1**. Cell movements during the direct co-culture system

**Supplementary Video S2**. Dynamic physical interactions between MDA-MB-231 and MCF10A cells

**Supplementary Video S3**. Time lapse imaging of MDA-MB-231 cell’s movements in vivo at one hour after inoculation.

**Supplementary Video S4**. Time lapse imaging of MDA-MB-231 cell’s movements in vivo at 24 hours after inoculation.
